# A Power-Frequency Electric Field Sensor for Portable Measurement

**DOI:** 10.3390/s18041053

**Published:** 2018-03-31

**Authors:** Dongping Xiao, Qichao Ma, Yutong Xie, Qi Zheng, Zhanlong Zhang

**Affiliations:** State Key Laboratory of Power Transmission Equipment & System Security and New Technology, Chongqing University, Chongqing 400044, China; 20151113002t@cqu.edu.cn (Q.M.); 20151102059t@cqu.edu.cn (Y.X.); 20161113031t@cqu.edu.cn (Q.Z.); zhangzl@cqu.edu.cn (Z.Z.)

**Keywords:** double spherical shell, performance requirements, portable measurement, power frequency electric field, sensor

## Abstract

In this paper, a new type of electric field sensor is proposed for the health and safety protection of inspection staff in high-voltage environments. Compared with the traditional power frequency electric field measurement instruments, the portable instrument has some special performance requirements and, thus, a new kind of double spherical shell sensor is presented. First, the mathematical relationships between the induced voltage of the sensor, the output voltage of the measurement circuit, and the original electric field in free space are deduced theoretically. These equations show the principle of the proposed sensor to measure the electric field and the effect factors of the measurement. Next, the characteristics of the sensor are analyzed through simulation. The simulation results are in good agreement with the theoretical analysis. The influencing rules of the size and material of the sensor on the measurement results are summarized. Then, the proposed sensor and the matching measurement system are used in a physical experiment. After calibration, the error of the measurement system is discussed. Lastly, the directional characteristic of the proposed sensor is experimentally tested.

## 1. Introduction

High-voltage equipment is always surrounded by a strong electric field, and studies have shown that power frequency electric fields have potential harmful effects on human health and safety [1,2,3]. An extremely high power frequency electric field will cause electrostatic discharge or even transient electric shock, and this will lead to other, more serious safety incidents. Therefore, some countries and international organizations have promulgated standards or recommended limitations regarding environmental power frequency electric field intensity for the general public and for power system staff [4,5,6,7]. However, power system practitioners are inevitably exposed to complex electric fields because they have to work close to high-voltage electrical equipment. If the practitioners can be equipped with a portable safety protection instrument that can measure the electric field intensity in the working area in real time and issue safety warnings, their safety and health could be ensured [8,9,10]. In this paper, a portable protection instrument is designed for the practitioners.

Power frequency electric field measurement is mainly governed by two principles: electromagnetic induction and optical effect. The electric field measurement instruments based on the Pockels optical effect include a laser generator, optical detector, photoelectric conversion, optical fiber, and other parts. There are more factors affecting measurement due to such a complex structure, leading to higher design complexity. Further, the movement of the human body will affect the incident angle of the laser, the internal stress of the optical fiber, and so on, which will change the phase delay of the laser, causing large measurement error. Thus, instruments based on optical effect are not suitable for portable measurement. The electric field measurement instruments based on capacitive sensing—a type of electromagnetic induction—are more widely used in power system environmental measurement because of their convenient manufacture, high accuracy, and strong overload capacity [11,12,13]. Therefore, an electric field sensor based on electromagnetic induction is discussed in this paper.

There are some commercialized electric field measurement instruments, such as the series of Narda, PMM, CA, etc., and a number of studies have continued to develop new electric field measurement instruments for special purposes [14,15,16,17]. These instruments are used mainly for electromagnetic environmental monitoring. During the measurement, the technicians are required to be far away from the probe braced with an insulated bracket. With the tedious steps in measuring the electric field, existing instruments cannot meet the requirements of portable measurement. Therefore, a new type of electric field measurement instrument is needed for special measuring purposes. In addition, the sensor is crucial to the portable electric field measurement.

A portable electric field sensor must meet the following performance requirements, which make it different from existing sensors: Requirement (1) is that the sensor should be small and light enough to wear on a person’s arm or waist. Requirement (2) is that the output of the sensor must be steady and accurate at a given measuring point even if the sensor is rotated. Requirement (3) is that the sensor must be suited to measure the complex power frequency electric field produced by varied electrical equipment in a power system.

There are cubical and spherical sensors [18,19,20,21], but both of them are unsuitable for portable measurement. There are three main reasons for this:

(1) The charges gather on the edge of electrodes, which causes electric field distortion in the adjacent space of the edges. The distortion is difficult to evaluate quantitatively, so it increases the uncertainty and instability of measurement.
(2) The sensors are too large and heavy for portable measurement. The size of the sensors is unlikely to be much reduced because the smaller the sensor size, the more serious the distortion caused by the edge effect.
(3) Because of their multi-electrode configuration, when the sensors are used for portable measurements, the mutual capacitances between the human body and these electrodes are extremely complicated [16], which will increase the uncertainty and instability of measurement.

After comparative studies on the existing sensor structures and measurement results, a new type of electric field sensor with double spherical shells is proposed to meet the requirements of portable measurement.

## 2. Structural Design of the Double Spherical Shell Sensor

**Figure 1:**
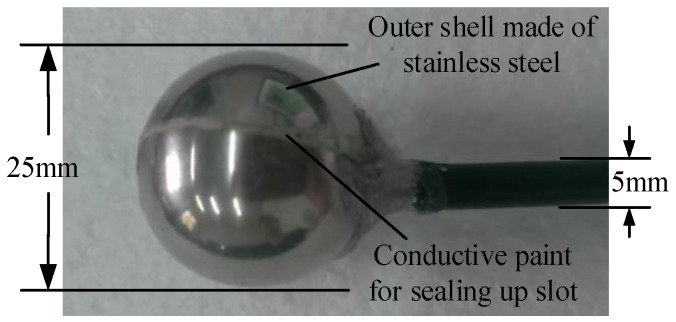
Picture of the double spherical shell sensor.

**Figure 2:**
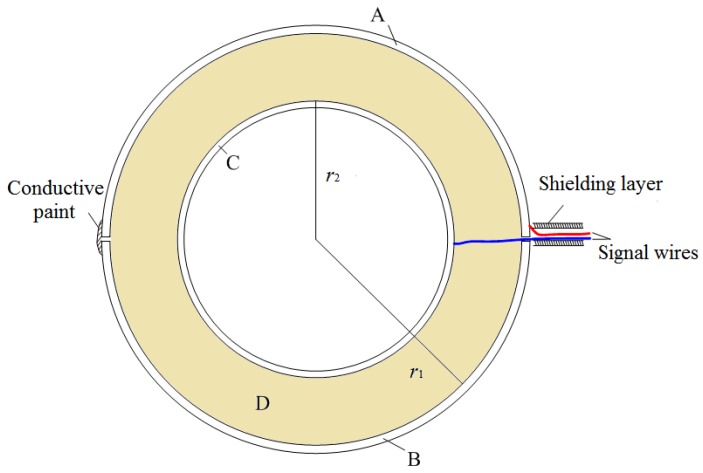
Structure of the double spherical shell sensor.

The double spherical shell sensor is shown in Figure 1, and the profile structure is shown in Figure 2.

The double spherical shell sensor is composed of the inner shell, the outer shell, and the fill medium. Parts A and B are two metal hemispherical shells with radius *r*_1_, the slot between which will be sealed up with conductive paint. Part C is a metal hollow shell with radius *r*_2_. Part D is a fill medium. The output signal of the sensor is the induced differential voltage *U*_AC_, which is extracted by shielded wire and connected to a differential amplifier. Besides this, the shielding layer of the shielded wire is connected to the reference ground of the signal processing circuit, which can eliminate the common-mode interference from the shielded cable in the electric field.

The inner and outer shells represent the two electrodes of the sensor. Each electrode is an entire spherical shell, enabling the sensor to increase in induction area and decrease in size. The inner electrode is hollow, which is conducive to reducing the weight. The measurement remains stable and the output value is fixed when the sensor is rotated because of the nearly symmetrical structure. Therefore, the requirements of portable measurement are all met.

## 3. Measurement Theory and Mathematical Models

### 3.1. Mathematical Models between Original Electric Field and Induced Voltage of Sensor

The induced charge will be generated and distributed on the surface of the outer shell when the sensor is placed in an electric field.

**Figure 3:**
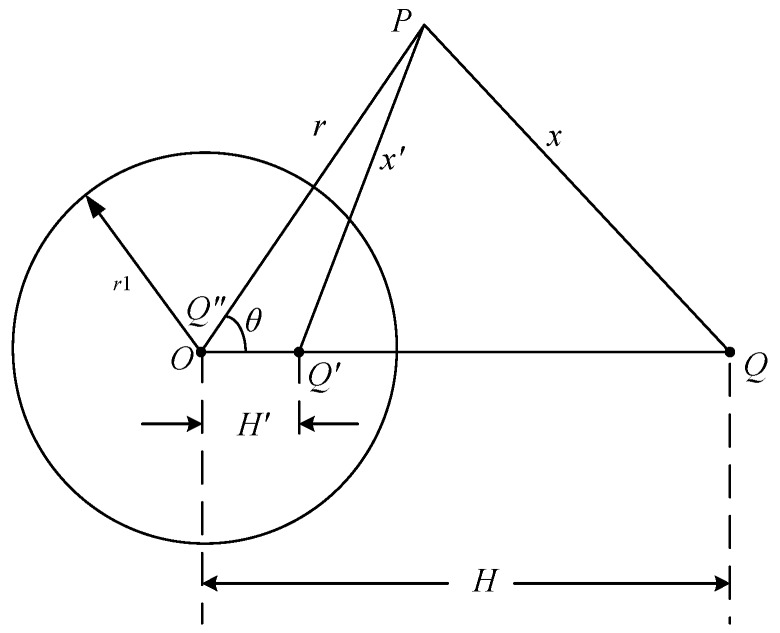
Sketch of calculation for the electric field.

To explain the measurement theory as a mathematical expression, we analyze the case that the electric field is generated by a point charge. The sketch of the calculation is shown in Figure 3. The spherical conducting shell with radius *r*_1_ is ungrounded and it is actually far away from the point charge.

Considering that the spherical shell is equipotential, the image charges *Q*′ and *Q*″ are set on the line linked by the center point *O* and point charge *Q* according to the uniqueness theorem and the image method. The position and value of the image charges are expressed as [22]

(1) $$Q^{\prime }=-\frac{r_{1}}{H}Q$$

(2) $$H^{\prime }=\frac{{r_{1}}^{2}}{H}$$

(3) $$Q^{\prime \prime }=-Q^{\prime }=\frac{r_{1}}{H}Q.$$

The potential at any point *P* in the space out of the outer shell is given by

(4) $$U=\frac{1}{4\pi \varepsilon_{0}}(\frac{Q}{x}+\frac{Q^{\prime }}{x^{\prime }}+\frac{Q^{\prime \prime }}{r})=\frac{Q}{4\pi \varepsilon_{0}}(\frac{1}{\sqrt{H^{2}+r^{2}-2Hr\cos\theta }}-\frac{r_{1}}{H\sqrt{H^{{}^{\prime }2}+r^{2}-2H^{\prime }r\cos\theta }}+\frac{r_{1}}{Hr})$$

Then the three components of electric field in the spherical coordinates are calculated by

(5) $$E_{1r}=-\frac{\partial U}{\partial r}=-\frac{Q}{4\pi \varepsilon_{0}}[\frac{-r+H\cos\theta }{{\left(H^{2}+r^{2}-2Hr\cos\theta \right)}^{3/2}}-\frac{r_{1}(r-H^{\prime }\cos\theta )}{H{\left(H^{{}^{\prime }2}+r^{2}-2H^{\prime }r\cos\theta \right)}^{3/2}}+\frac{r_{1}}{Hr^{2}}]$$

(6) $$E_{1\theta }=-\frac{1}{r}\frac{\partial U}{\partial \theta }=-\frac{Q}{4\pi \varepsilon_{0}r}[\frac{-Hr\sin\theta }{{\left(H^{2}+r^{2}-2Hr\cos\theta \right)}^{3/2}}+\frac{r_{1}H^{\prime }r\cos\theta }{H{\left(H^{{}^{\prime }2}+r^{2}-2H^{\prime }r\cos\theta \right)}^{3/2}}]$$

(7) $$E_{1\varphi }=-\frac{1}{r\sin\theta }\frac{\partial U}{\partial \varphi }=0.$$

Setting *r* = *r*_1_ as *r*_1_ << *H*, the electric field on the outer surface of shell can be calculated by bringing Equation (2) into Equations (5)–(7):

(8) $$\begin{array}{l} {E_{1r}|}_{r=R_{1}}=-\frac{Q}{4\pi \varepsilon_{0}}\frac{1}{r_{1}}\left(\frac{1}{H}-\frac{1}{x}\right)\\ x=\sqrt{H^{2}+{r_{1}}^{2}-2Hr_{1}\cos\theta } \end{array}$$

(9) $${E_{1\theta }|}_{r=r_{1}}=0$$

(10) $${E_{1\varphi }|}_{r=r_{1}}=0.$$

The electric field on the outer surface of the shell is perpendicular to the surface. The field intensity changes with *x*, and it reaches the maximum when *x* = *H* − *r*_1_, while it is equal to zero when *x* = *H*.

If there is no spherical shell in the space, the original electric field generated by the same point charge along the virtual circle shown in Figure 3 is

(11) $$E_{0}=\frac{Q}{4\pi \varepsilon_{0}}\frac{1}{H^{2}+{r_{1}}^{2}-2Hr_{1}\cos\theta }\approx \frac{Q}{4\pi \varepsilon_{0}}\frac{1}{H^{2}}.$$

If the material of the shells is not a perfect conductor and the electric field is an alternating field, the electric field cannot be shielded completely by the outer shell; this results in the existence of an electric field *E*_2*r*_ between the outer and inner shells. Because of the spherically symmetric structure of the sensor, *E*_2*r*_ is a spherically symmetric distribution between the two shells. The radial component *E*_2*r*_ can be expressed as

(12) $$E_{2r}=\frac{r_{1}^{2}}{r^{2}}{E_{2r}|}_{r=r_{1}}$$

where ${E_{2r}|}_{r=r_{1}}$ is the electric field intensity on the inner surface of the outer shell.

The induced voltage *U*_AC_ is calculated by the integral of the electric field

(13) $$U_{\mathrm{AC}}=\int_{r_{1}}^{r_{2}}-E_{2r}dr=\frac{r_{1}\left(r_{1}-r_{2}\right)}{r_{2}}{E_{2r}|}_{r=r_{1}}.$$

Since *E*_2_ is spherically symmetric, no matter what the field distribution on the surface of the outer shell is, ${E_{2r}|}_{r=r_{1}}$ can be expressed as

(14) $${E_{2r}|}_{r=r_{1}}=K_{1}E_{0}.$$

The coefficient *K*_1_ is related to the radius, thickness, and conductivity of the outer shell, the conductivity of the fill medium, and the frequency of the original electric field.

Substituting Equation (14) into Equation (13), the induced voltage *U*_AC_ and the original electric field *E*_0_ are in the following relationship:

(15) $$U_{\mathrm{AC}}=\frac{K_{1}r_{1}\left(r_{1}-r_{2}\right)}{r_{2}}E_{0}=K_{2}E_{0}.$$

The value of the coefficient *K*_2_ is obtained by simulation in Section 4.

### 3.2. Mathematical Models between Induced Voltage of Sensor and Output of Signal Extraction Module

**Figure 4:**
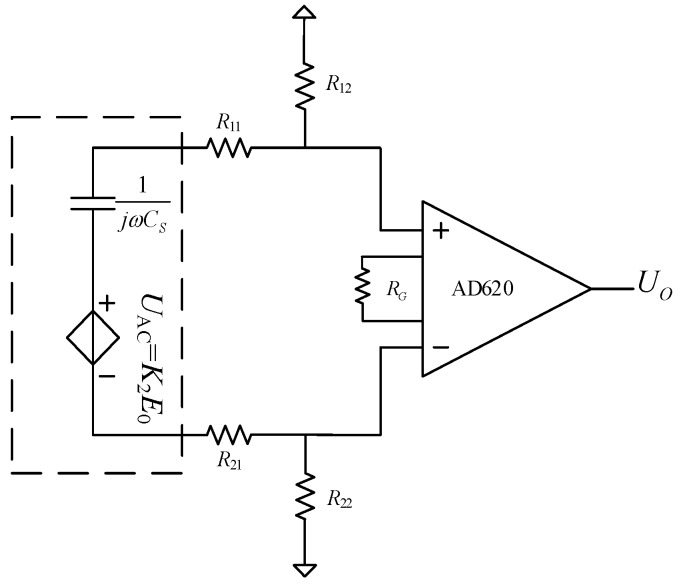
Equivalent circuit of the signal extraction module.

The potential of the shells is a floating potential, and the output signal of the sensor is the differential potential of the two shells. A differential amplifier is needed in the signal extraction module, as shown in Figure 4. AD620 is a low-cost and high-accuracy instrumentation differential amplifier, which can eliminate the common-mode interference due to electromagnetic induction on the signal wires and amplify *U*_AC_.

In Figure 4, the induced voltage is equivalent to an alternating voltage source *U*_AC_ with angular frequency *ω*. *C_S_* is the inherent capacitance of the shells, and can be calculated by

(16) $$C_{S}=\frac{4\pi \varepsilon r_{1}r_{2}}{r_{1}-r_{2}}.$$

*R*_11_ and *R*_21_ are the current-limiting resistors. *R*_22_ and *R*_12_ are the DC bias resistors. The AD620 requires only one external resistor *R_G_* to set the gain *G* from 1 to 10,000.

(17) $$G=\frac{49.4k\Omega }{R_{G}}+1$$

Considering that the ports “+” and “−” are connected to operational amplifiers in the chip AD620, the current flow into the two ports is zero. Thus, the components *C_S_*, *R*_11_, *R*_12_, *R*_22_, and *R*_21_ form a loop, and the output voltage *U_O_* can be obtained as follows.

(18) $$U_{O}=G\frac{R_{12}+R_{22}}{\left(R_{11}+R_{21}+R_{12}+R_{22}\right)+\frac{1}{j\omega C_{S}}}\cdot U_{\mathrm{AC}}=G\frac{j\omega C_{S}\left(R_{12}+R_{22}\right)}{j\omega C_{S}\left(R_{11}+R_{21}+R_{12}+R_{22}\right)+1}\cdot U_{\mathrm{AC}}$$

Further, the RMS value of *U_O_* can be expressed as follows.

(19) $$U_{O\_\mathrm{rms}}=G\frac{\omega C_{S}\left(R_{12}+R_{22}\right)}{\sqrt{\omega^{2}C_{S}^{2}{\left(R_{11}+R_{21}+R_{12}+R_{22}\right)}^{2}+1}}\cdot U_{\mathrm{AC}\_\mathrm{rms}}=K_{3}U_{\mathrm{AC}\_\mathrm{rms}}$$

Combining Equation (15) with Equation (19), the RMS value *E*_0_rms_ can be expressed as

(20) $$E_{0\_\mathrm{rms}}=\frac{1}{K_{2}K_{3}}U_{O\_\mathrm{rms}}=K_{all}U_{O\_\mathrm{rms}}.$$

Therefore, the electric field *E*_0_rms_ in the free space can be determined by the measurement voltage *U_O_*__rms_, and the coefficient *K_all_* is obtained and verified by experiment in Section 5.

## 4. Simulation and Discussion

### 4.1. Modeling and Results of Simulation

A 3-D simulation model was built based on the software Ansoft/Maxwell [23]. The radii of the outer and inner shells were *r*_1_ = 12.5 mm and *r*_2_ = 8 mm, respectively. The material of the shells was stainless steel, and the fill medium was epoxy resin. The sensor was placed in a homogeneous electric field, which was generated by a wide parallel plate capacitor.

**Figure 5:**
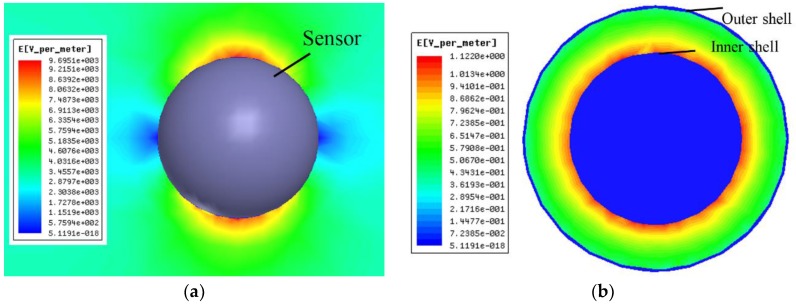
The distribution of electric field. (**a**) Around the sensor. (**b**) Inside the sensor.

A sinusoidal voltage with power frequency of 50 Hz and adjustable amplitude was set on the parallel plates. When the original electric field between the plates was set as 3 kV/m, the distribution of electric field in the outer and inner parts of the sensor were as shown in Figure 5.

Figure 5a shows that the electric field varies regularly along the outer surface of the outer shell, which is consistent with the theoretical analysis. Figure 5b shows that there is an electric field between the shells, which causes potential difference between the two shells.

**Figure 6:**
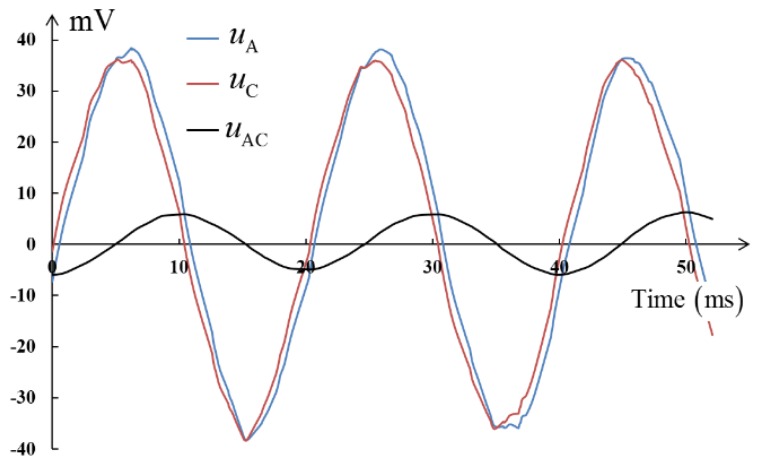
Time domain waveform of voltage.

The time domain waveforms of the outer shell potential *u*_A_, the inner shell potential *u*_C_, and the potential difference, i.e., the induced voltage *u*_AC_, are shown in Figure 6.

According to Figure 6, the waveform of *u*_AC_ is a sine wave with frequency 50 Hz and the effective value is approximately 4.28 mV.

The intensity of the original electric field *E*_0_rms_ is changed, and the corresponding induced voltages *U*_AC_rms_ are shown in Table 1.

Analyzing the data above, the linearly dependent coefficient between *E*_0_rms_ and *U*_AC_rms_ is close to 1.00, which means that *U*_AC_rms_ has a significant positive relationship with *E*_0_rms_. Therefore, the induced voltage can be regarded as being proportional to the original electric field. The proportionality coefficient between *E*_0_rms_ and *U*_AC_rms_ is defined as *K*_2_ in Equation (15), and *K*_2_ ≈ 1.42 × 10^−6^ m.

### 4.2. Analysis of Effect Factors of Induced Voltage

#### 4.2.1. Effect of Sensor’s Size

The original electric field was fixed at 3 kV/m. The materials of shells and fill medium were stainless steel and epoxy resin, respectively. The radii of the outer shell *r*_1_ and inner shell *r*_2_ were changed, and the corresponding induced voltages *U*_AC_rms_ are shown in Table 2.

Based on Table 2, some conclusions can be drawn. First, when the inner radius is fixed, a smaller outer radius results in weaker induced voltage. Second, when the outer radius is fixed, a smaller inner radius results in stronger induced voltage. Finally, when the distance between the inner and outer shells is fixed, the induced voltage becomes stronger as the outer radius decreases.

The size of the sensor affects the magnitude of induced voltage and the fabrication complexity of the sensor. The requirements of small size and light weight should also be considered. Thus, the size of sensor in this paper was chosen as *r*_1_ = 12.5 mm and *r*_2_ = 8 mm.

#### 4.2.2. Effect of Shells’ Material

Only metal material was taken into account because the shells must have equipotential character. In the simulation, the original electric field was fixed at 3 kV/m, and the material of the shells ranged over stainless steel, iron, aluminum, copper, and perfect conductor. The spacer material was epoxy resin, and the thickness of the two shells was set as 0.5 mm. The corresponding induced voltages *U*_AC_rms_ are shown in Table 3.

It can be seen from Table 3 that there will be no electric field between the two shells if the material is a perfect conductor, because the shielding effectiveness of the perfect conductor is ∞. However, the material cannot, in fact, be a perfect conductor. The lower conductivity of the material leads to the lower shielding effectiveness, and a stronger electric field between the two shells. Therefore, stainless steel was chosen as the shells’ material. The stainless steel shells are still equipotential in an electric field.

#### 4.2.3. Effect of Filling Material

The filling material between the two shells ranged over vacuum, epoxy resin, glass, and mica. The corresponding induced voltages *U*_AC_rms_ are shown in Table 4.

It can be seen from Table 4 that the induced voltage of the sensor decreases with the increase of the relative dielectric constant of the filling material. Considering that the fill medium is also used to support the two shells, the spacer material cannot be vacuum or air. In this paper, epoxy resin was chosen as the spacer material, because the output voltage can be as strong as possible and epoxy resin is easy to mold. The bulk conductivity of epoxy resin is 1 × (10^−15^~10^−13^) S/m, and the dielectric loss tangent (tgδ) is less than 0.004.

## 5. Experiment and Discussion

### 5.1. Overall Design of the Portable Electric Field Measurement System and Experiment Environment

**Figure 7:**
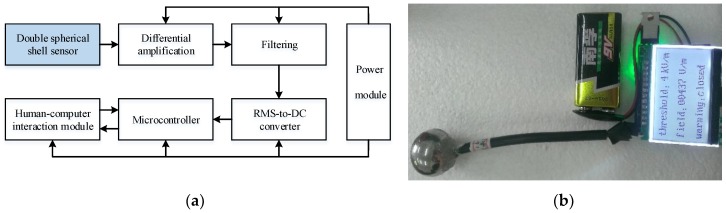
Overall design of the portable electric field measurement system. (**a**) Structure diagram. (**b**) Physical photograph.

The designed portable electric field sensor and measurement system are shown in Figure 7.

The radii of the outer and inner shell were 12.5 mm and 8 mm, respectively, so the inherent capacitance of the shells can be calculated as 8.897 pF according to Equation (16). The external resistor *R*_G_ of AD620 was set as 100 Ω, so the gain G can be calculated as 495 according to Equation (17). In Figure 4, the resistors *R*_11_ and *R*_21_ are set as 1 kΩ, and *R*_22_ and *R*_12_ are set as 50 MΩ, so the coefficients *K*_3_ and *K_all_* can be calculated as 133 and 5285 m^−1^, according to Equations (19) and (20), respectively. The value of *K_all_* will be verified by experiment.

**Figure 8:**
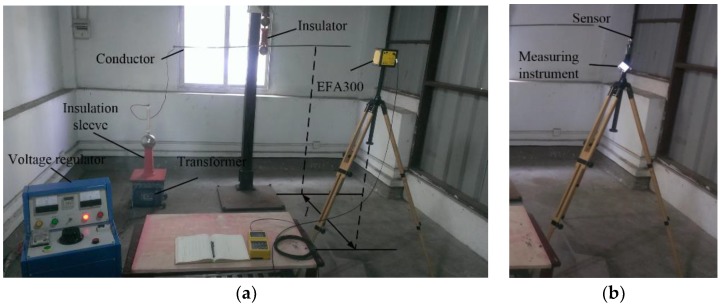
Experimental environment. (**a)** Using EFA-300. (**b**) Using the designed instrument.

In order to study the characteristic of the instrument in an inhomogeneous electric field, an experimental platform was built as shown in Figure 8. The platform was composed of a voltage regulator, a transformer, a conductor, an insulation sleeve, and two electric field measurement instruments. The input voltage of the voltage regulator was AC 220 V and the output voltage could be adjusted from 0 to AC 250 V. The ratio of the boosting transformer was 200:1. Thus, the voltage on the conductor could be adjusted from 0 to 50 kV, and electric fields with different intensity were generated in space. EFA-300 is one of the widely used electromagnetic field measurement instruments produced by Narda. The measurement frequency range of the EFA-300 is 5 Hz–32 kHz, and the measurement range is 0.7 V/m–100 kV/m; the noise floor is only 0.45 V/m [24]. In the experiments, the measurement results of the EFA-300 are considered as the original electric field for data comparison and calibration.

### 5.2. Calibration and Measurement Error Analysis of the Sensor

To calculate the proportionality coefficient *K_all_* as shown in Equation (20) by experiment, a series of experiments were carried out.

**Figure 9:**
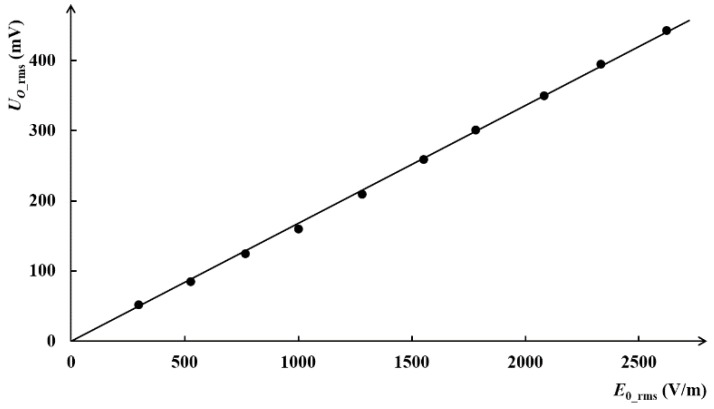
The measuring results and fitting curve.

Firstly, the measurement position was fixed, and electric fields in space with different intensity were generated by setting different excitation voltages on the conductor. The measurement data of *E*_0_rms_ and *U_O_*__rms_ are shown in Table 5, and the fitting curve of them is plotted in Figure 9.

It is clear that *U_O_*__rms_ is proportional to *E*_0_rms_. According to the fitting curve, the proportionality coefficient is calculated as *K*_all_ = 5950 m^−1^, which is closed to the theoretical value (5285 m^−1^).

**Figure 10:**
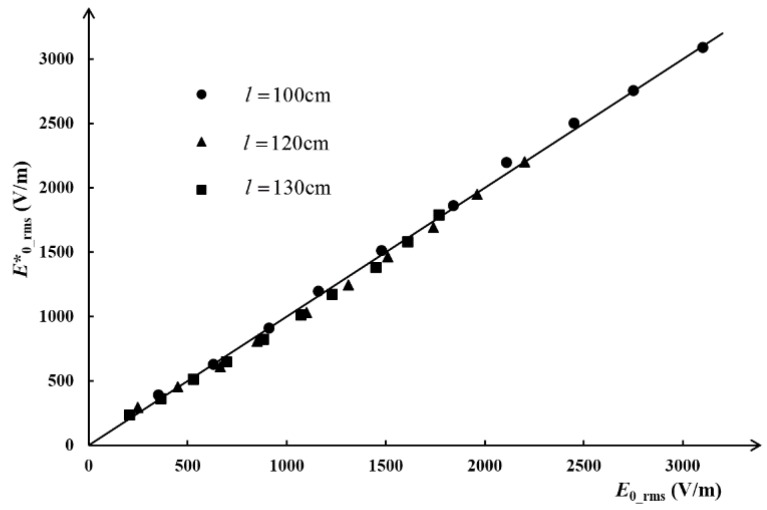
The results in different measuring positions.

To confirm that the coefficient is valid when the measurement position is changed, we set three kinds of vertical distance (*l*) between the conductor and the sensor, and accordingly performed three sets of experiments to obtain *U_O_*__rms_ under a series of electric fields *E*_0_rms_. Then, *U_O_*__rms_ multiplied by 5950 m^−1^ gives E^*^_0_rms_ which represents the measuring electric field obtained by the designed instrument. The correlation between E^*^_0_rms_ and *E*_0_rms_ is shown in Figure 10.

In Figure 10, any point that falls on the reference line means E^*^_0_rms_ = *E*_0_rms_. It is clear that most of the points fall on the reference line or are very close to the line no matter what kind of measurement distance or original electric field intensity was set. The absolute error is less than ±50 V/m with standard deviation 25 V/m, no matter the original electric field strength. The maximum relative error is 11%, which appears when the electric field is about 247 V/m, and it was found by further analysis that the relative error would be less than 5% when the original electric field is stronger than 1 kV/m. The reason for this is that the stronger the original electric field, the greater the signal-to-noise ratio. Considering the fact that the proposed electric field sensor and measurement system are designed for use in strong electric field environments, and a weak electric field will not trigger the alarm of the instrument, this error in measurement is acceptable.

### 5.3. Experiment for Directional Characteristics of the Sensor

Theoretically speaking, the output voltage will not change when the designed sensor is rotated in the same electric field because of the symmetrical structure. This section presents the directional performance test of the instrument.

**Figure 11:**
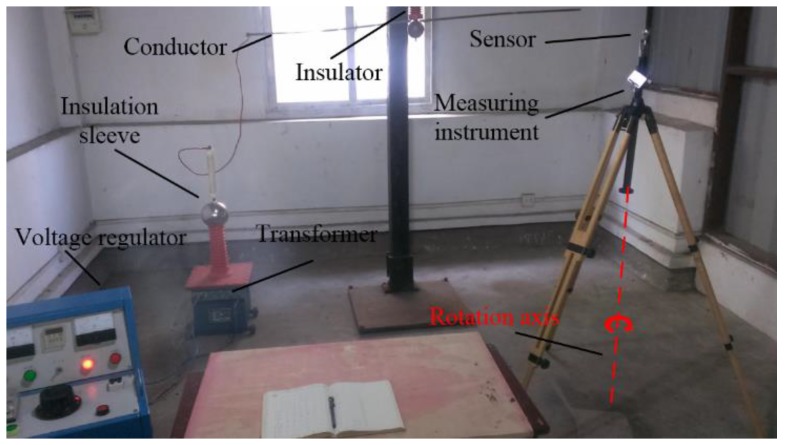
Experiment for testing the directional characteristic of the sensor.

The center position of the instrument was fixed, and the measuring system was rotated along the vertical axes, as shown in Figure 11.

The measurement results are shown in Table 6.

Table 6 shows that the mean value of measurement results remains 2202 V/m to 2345 V/m when the instrument is rotated, which shows the measurement stability of the instrument. The above results indicate that the measuring results will be stable when the instrument is rotated by the carrier.

## 6. Conclusions

Portable electric field measurement is a new kind of demand, at the core of which is the need to design a new sensor that meets specific performance requirements. This paper presented a new type of sensor with a double spherical shell. The mathematical relationships between output voltage of the sensor, measuring voltage of the measurement circuit, and original electric field were derived in theory. The analysis demonstrated the feasibility of the sensor and the factors that affect the measurement.

The characteristic of the electric field sensor was simulated in a homogeneous field. The output voltage of the sensor is mainly affected by the radii of outer and inner shells as well as the material used for the shells and fill medium. Considering the requirements of small size, light weight, and convenient processing, the radii of the outer and inner shells were set at 12.5 and 8 mm, respectively, and stainless steel and epoxy resin were chosen as the material of the shells and fill medium, respectively.

The experiment was conducted in an inhomogeneous field generated by a conductor. The results demonstrate the high accuracy of the sensor, with absolute error less than 50 V/m, and relative error less than 5% when the original electric field is stronger than 1 kV/m. Furthermore, when the double spherical shell sensor is rotated along the axes but the center position remains fixed, the output voltage remains almost unchanged, which is what is required for portable measurement.

## Figures and Tables

**Table 1:** *U*_AC_rms_ obtained under different *E*_0_rms_.

*E*_0___rms_ (kV/m)	1	2	3	4	5	6	7	8	9	10
*U*_AC___rms_ (mV)	1.43	2.85	4.28	5.71	7.13	8.57	10.01	11.30	12.84	14.20

**Table 2:** *U*_AC_rms_ (mV) obtained by setting different sensor sizes.

	*r*_1_ (mm)	9.5	11	12.5	14	15.5
*r*_2_ (mm)						
8		0.53	2.13	4.28	6.73	9.54
9.5		—	0.44	1.74	3.77	5.73
11		—	—	0.37	1.56	3.36
12.5		—	—	—	0.33	1.41
14		—	—	—	—	0.29

**Table 3:** *U*_AC_rms_ obtained by setting different shell materials.

Material	Conductivity (S/m)	*U*_AC_rms_ (mV)
stainless steel	1.1 × 10^6^	4.28
iron	1.03 × 10^7^	3.40
aluminum	3.8 × 10^7^	2.90
copper	5.8 × 10^7^	0.83
perfect conductor	1 × 10^30^	0

**Table 4:** *U*_AC_rms_ obtained by setting different filling materials.

Material	Relative Dielectric Constant	*U*_AC_rms_ (mV)
vacuum	1	13.44
epoxy resin	3.6	4.28
glass	5.5	2.32
mica	5.7	2.31

**Table 5:** Statistical data of *E*_0_rms_ and *U_O_*__rms_ when fixing measurement point.

*E*_0___rms_ (V/m)	297	525	767	1000	1280
*U_O_*__rms_ (mV)	52	85	120	160	210
*E*_0___rms_ (V/m)	1550	1780	2080	2330	2620
*U_O_*__rms_ (mV)	259	301	350	395	443

**Table 6:** Results of the experiment for directional characteristics of the sensor.

Angle	0°	45°	90°	135°	180°	225°	270°	315°
E^*^_0___rms_ (V/m)	2262	2220	2333	2202	2286	2315	2345	2274
